# Disease mapping of early- and late-stage cancer to monitor inequalities in early detection: a study of cutaneous malignant melanoma

**DOI:** 10.1007/s10654-020-00637-0

**Published:** 2020-04-30

**Authors:** Ulf Strömberg, Brandon L. Parkes, Anders Holmén, Stefan Peterson, Erik Holmberg, Amir Baigi, Frédéric B. Piel

**Affiliations:** 1 School of Public Health and Community Medicine, Institute of Medicine, Sahlgrenska Academy At University of Gothenburg, PO Box 463, 405 30 Gothenburg, Sweden; 2 Department of Research and Development, Region Halland, Halmstad, Sweden; 3 UK Small Area Health Statistics Unit (SAHSU), Department of Epidemiology and Biostatistics, School of Public Health, Imperial College, London, UK; 4 Regional Cancer Centre South, Lund, Sweden; 5 Regional Cancer Centre West, Gothenburg, Sweden; 6 National Institute for Health Research Health Protection Research Unit (NIHR HPRU) in Health Impact of Environmental Hazards, Imperial College London, London, UK

**Keywords:** Early detection of cancer, Epidemiological monitoring

## Abstract

We consider disease mapping of early- and late-stage cancer, in order to identify and monitor inequalities in early detection. Our method is demonstrated by mapping cancer incidence at high geographical resolution using data on 10,302 cutaneous malignant melanoma (CMM) cases within the 3.7 million population of South-West Sweden. The cases were geocoded into small-areas, each with a population size between 600 and 2600 and accessible socio-demographic data. Using the disease mapping application Rapid Inquiry Facility (RIF) 4.0, we produced regional maps to visualise spatial variations in stage I, II and III–IV CMM incidences, complemented by local maps to explore the variations within two urban areas. Pronounced spatial disparities in stage I CMM incidence were revealed by the regional and local maps. Stage I CMM incidence was markedly higher in wealthier small-areas, in particular within each urban area. A twofold higher stage I incidence was observed, on average, in the wealthiest small-areas (upper quintile) than in the poorest small-areas (lower quintile). We identified in the regional map of stage III–IV CMM two clusters of higher or lower than expected late-stage incidences which were quite distinct from those identified for stage I. In conclusion, our analysis of CMM incidences supported the use of this method of cancer stage incidence mapping for revealing geographical and socio-demographic disparities in cancer detection.

**Electronic supplementary material:**

The online version of this article (10.1007/s10654-020-00637-0) contains supplementary material, which is available to authorized users.

## Introduction

Efforts to detect cancer early are considered crucial for reducing the burden of these progressive diseases. Monitoring progress towards better prevention relies on the availability of detailed stage-level data, which remains patchy in most high-income countries. In a population‐based international comparisons of survival by stage, the International Cancer Benchmarking Partnership identified marked discrepancies in stage data management and in the classifications used, both between countries and over time, while acknowledging that the collection of staging data needed to be balanced to meet both clinical and epidemiological demands [1]. Some countries, such as Scotland in 2012, have adopted an early detection programme aiming at increasing the proportion of people diagnosed with stage I cancer [2]. Although prevention initiatives can result in substantial improvements in detection uptake, their evaluation based on counts (number of cases) stratified by tumour stage at diagnosis, as reported, for example, from Scotland [2], Sweden [3] and Denmark [4], needs to be carefully conducted. Increasing the number of patients diagnosed with early-stage tumours may at first appear as a successful outcome of prevention programmes. However, consider an ‘overdiagnosis scenario’ [5] where the incidence of late-stage cancer in a population group is not lowered despite the cases show a declining proportion with late-stage tumours. Monitoring based on proportion of cases with late-stage cancer (or, conversely, early-stage cancer) may hamper correct inference. In addition, such evaluations can be confounded by various factors, including age and socio-economic status. For example, studies of differences in counts can be biased if not adjusted for age when higher age is associated with later stage at diagnosis and the age distribution differs between patient groups. It is therefore important to ensure that assessment measures used are comparable over space and time.

Disease mapping is a widely used method for analysing spatially linked data. It offers the advantage of presenting a detailed view of the study area with relevant information about the estimated outcomes. Age and sex standardisation can help avoiding confounding due to demographic factors, while Bayesian spatial smoothing and adjusting for socio-demographics inequalities can improve the reliability of estimates and facilitate comparisons between areas [6]. Disease mapping of all-stage cancer incidence is a ‘state-of-the-art’ procedure [7]. Herein, we focus on mapping of early- and late-stage cancer at a high geographical resolution for analysing inequalities in early detection. Besides helping to identify geographical disparities in stage-specific incidences, disease mapping may provide results that reveal links between small-area deprivation and stage-specific incidences. Potentially, this approach can provide crucial knowledge for rational targeting of public health interventions to improve early detection.

We suggest a disease-mapping approach based on stage-specific incidences, rather than stage-proportions of cases, to more objectively monitor progress towards cancer prevention and to identify disparities in early detection of cancer. We chose to demonstrate this approach using data on cutaneous malignant melanoma (CMM) from the Southern and Western Health Care Regions of Sweden (population size: 3.66 million in 2016; 37% of the national population). CMM is the fifth most common cancer type in Sweden [8] and, as in other western countries [9], its incidence has increased during the last decades. Furthermore, stage at diagnosis has been shown to be a valuable prognostic factor in this population [10].

## Methods

### Data sources

#### CMM cases

All individuals, aged 30 or older, residing in the Southern and Western Swedish Health Care Regions and diagnosed with a first invasive CMM between January 1, 2008, and December 31, 2016, were considered eligible. Data on the sex, age and year of diagnosis of all cases were extracted from the Swedish Cancer Registry [11]. Registry holders in Sweden use the unique Swedish personal numbers for data linkage [12]. Information on the tumour stage for each case was obtained through linkage with the Swedish Melanoma Registry (> 98% coverage) [13]. The following definitions were used [14]:

- Stage I: all CMMs with a Breslow thickness ≤ 1.0 mm and CMMs with a 1.1–2.0 mm thickness without ulceration (early detection);
- Stage II: CMMs of 1.1–2.0 mm thickness with ulceration and all CMMs > 2.0 mm;
- Stage III-IV (combined due to the rarity of stage IV): CMMs with spreading to regional lymph nodes (III) or with distant metastases (IV).

#### Small areas and small-area-level data

Residential small-areas were delimited according to Demographic Statistics Areas (DSAs). DSAs are new geographical boundaries, defined by population size and key features (e.g. streets, waterways and railways). They were launched in 2018 by Statistics Sweden with the aim to facilitate monitoring of segregation and socio-economic conditions in small geographic areas [15].

The geocoding of each case to a residential small-area at the year of diagnosis (data extracted at year end) was performed by Statistics Sweden. Population data by sex and 5-year age groups for each year in the study period (2008–2016) were obtained data from Statistics Sweden’s population registers [16], alongside yearly, aggregated data on small-area socio-demographic characteristics [17]. We used the median income per household per consumption unit as our indicator of small-area deprivation. We found high correlations (*r* > 0.70) between this variable and other variables of deprivation reflecting educational level, unemployment and purchasing power. The DSAs in each of the study areas were categorised into quintiles of the median income values (Q1 = poorest to Q5 = wealthiest; Table 1).

**Table 1:** Stage at diagnosis for the 10,302 cases of cutaneous malignant melanoma (CMM) included in this study, together with statistics based on the proportions of cases with late-stage tumours (stage II–IV and stage III–IV diagnoses, respectively)

	Stage I CMM cases across age groups and quintiles of small-area deprivation		Stage II CMM cases across age groups and quintiles of small-area deprivation		Stage III–IV CMM cases across age groups and quintiles of small-area deprivation		Statistics based on proportion of cases with stage II–IV tumours (observed proportions and associations with age and small-area deprivation)			Statistics based on proportion of cases with stage III–IV tumours (observed proportions and associations with age and small-area deprivation)		
	Number	(%)	Number	(%)	Number	(%)	Proportion	OR^a^	(95% CI)	Proportion	OR^b^	(95% CI)
Age group (years)												
30– 34	254	(3.5)	27	(1.2)	16	(2.2)	0.14	1.0	Ref.	0.05	1.0	Ref.
35–39	396	(5.4)	43	(1.9)	29	(4.0)	0.15	1.07	(0.71–1.61)	0.06	1.16	(0.62–2.17)
40–44	538	(7.4)	59	(2.6)	36	(5.0)	0.15	1.05	(0.71–1.56)	0.06	1.03	(0.56–1.89)
45–49	599	(8.2)	79	(3.5)	45	(6.2)	0.17	1.22	(0.84–1.78)	0.06	1.14	(0.63–2.05)
50–54	592	(8.1)	108	(4.8)	50	(6.9)	0.21	1.56	(1.08–2.26)	0.07	1.23	(0.69–2.20)
55–59	717	(9.8)	139	(6.1)	82	(11.3)	0.24	1.77	(1.24–2.54)	0.09	1.63	(0.94–2.84)
60–64	818	(11.2)	193	(8.5)	73	(10.0)	0.25	1.86	(1.31–2.65)	0.07	1.17	(0.67–2.05)
65–69	1009	(13.8)	256	(11.3)	98	(13.5)	0.26	1.97	(1.39–2.78)	0.07	1.26	(0.73–2.17)
70–74	837	(11.5)	298	(13.1)	98	(13.5)	0.32	2.68	(1.89–3.79)	0.08	1.43	(0.83–2.47)
75–79	685	(9.4)	298	(13.1)	90	(12.4)	0.36	3.15	(2.22–4.46)	0.08	1.52	(0.88–2.64)
80–84	472	(6.5)	323	(14.2)	61	(8.4)	0.45	4.52	(3.18–6.42)	0.07	1.28	(0.73–2.26)
85–89	290	(4.0)	270	(11.9)	28	(3.9)	0.51	5.72	(4.98–8.22)	0.05	0.84	(0.45–1.58)
90 +	95	(1.3)	180	(7.9)	21	(2.9)	0.68	11.8	(7.86–17.7)	0.07	1.21	(0.61–2.40)
Total	7302		2273		727							
Small-area deprivation within the study region, by median income quintiles (thousands of SEK/year)												
Q1 (108–189)	973	(13.3)	418	(18.4)	110	(15.1)	0.35	1.40^c^	(1.21–1.62)	0.07	1.07^c^	(0.83–1.37)
Q2 (190–212)	1362	(18.7)	490	(21.6)	139	(19.1)	0.32	1.27^c^	(1.11–1.45)	0.07	1.00^c^	(0.79–1.26)
Q3 (213–230)	1457	(20.0)	469	(20.6)	159	(21.9)	0.30	1.30^c^	(1.13–1.48)	0.08	1.12^c^	(0.89–1.39)
Q4 (231–252)	1574	(21.6)	447	(19.7)	140	(19.3)	0.27	1.15^c^	(1.00–1.32)	0.06	0.92^c^	(0.73–1.16)
Q5 (253–398)	1936	(26.5)	449	(19.8)	179	(24.6)	0.24	1.0	Ref.	0.07	1.0	Ref.
Small-area deprivation within Gothenburg, by median income quintiles (thousands of SEK/year)												
Q1 (123–179)	61	(6)	41	(13)	8	(11)	0.45	2.95^c^	(1.85–4.69)	0.07	1.90^c^	(0.79–4.56)
Q2 (180–208)	186	(18)	59	(19)	17	(24)	0.29	1.52^c^	(1.05–2.20)	0.06	1.70^c^	(0.85–3.39)
Q3 (209–240)	192	(19)	74	(24)	13	(18)	0.31	1.82^c^	(1.27–2.61)	0.05	1.17^c^	(0.56–2.44)
Q4 (241–272)	234	(23)	61	(20)	15	(21)	0.25	1.23^c^	(0.85–1.76)	0.05	1.18^c^	(0.58–2.39)
Q5 (273–393)	344	(34)	71	(23)	18	(25)	0.21	1.0	Ref.	0.04	1.0	Ref.
Small-area deprivation within Malmoe, by median income quintiles (thousands of SEK/year)												
Q1 (108–162)	24	(6)	23	(15)	7	(12)	0.56	2.71^c^	(1.42–5.15)	0.13	1.42^c^	(0.55–3.70)
Q2 (163–192)	67	(16)	27	(18)	12	(20)	0.37	1.33^c^	(0.78–2.25)	0.11	1.30^c^	(0.59–2.87)
Q3 (193–217)	85	(20)	29	(19)	6	(10)	0.29	0.90^c^	(0.54–1.53)	0.05	0.55^c^	(0.21–1.43)
Q4 (218–246)	111	(26)	35	(23)	15	(25)	0.31	0.98^c^	(0.61–1.58)	0.09	1.01^c^	(0.49–2.09)
Q5 (247–398)	134	(32)	36	(24)	19	(32)	0.29	1.0	Ref.	0.10	1.0	Ref.

Small-area deprivation is defined by median income quintiles in thousands of Swedish krona per year independently for each of the three study areas considered: the Southern and Western Swedish Health Care Regions, and the municipalities of Gothenburg and Malmoe

*SEK* Swedish krona

^a^Odds ratios (ORs) with 95% confidence intervals (CIs) obtained from multivariable logistic regression model of age, sex and residential small-area (according the deprivation classification) on the odds of having a stage II-IV diagnosis. OR for sex not given in table [men vs. women, 1.18 (1.07–1.29)]

^b^Odds ratios (ORs) with 95% confidence intervals (CIs) obtained from multivariable logistic regression model of age, sex and residential small-area (according the deprivation classification) on the odds of having a stage III–IV diagnosis. OR for sex not given in table [men vs. women, 1.30 (1.11–1.52)]

^c^The age- and sex- adjusted ORs with regard to small-area deprivation within the two urban areas Gothenburg and Malmoe, respectively

We conducted two sets of analyses: one for the Southern and Western Swedish Health Care Regions (regional level), and another for main urban areas within the regions (local level). An overview of the study area and the spatially linked data is presented in Supplementary Fig. S1. Our regional mapping covered the 2173 small-areas (DSAs) within the Southern and Western Swedish Health Care Regions. Each DSA had a mean population size (considering the observation period 2008–2016) ranging between 600 and 2600. Our local mappings were based on data from each of the two most densely populated urban areas: Gothenburg (306 DSAs) and Malmoe (192 DSAs).

### Analysis of proportion of cases with late-stage tumours

Based on the case data set solely, we used a logistic regression model for estimating associations between age, sex and residential small-area (according a deprivation classification) of each CMM case and the odds of having a late-stage diagnosis. We considered two alternative definitions of late-stage diagnosis: (i) stage II–IV disease at diagnosis and (ii) stage III–IV disease at diagnosis. We reinforce that this analysis was carried out to demonstrate the potential caveats in methodologies commonly applied to assess inequalities in early detection of cancer.

### Analysis of stage-specific incidences

#### Overall time-trends

Time trends of stage-specific incidences are presented for the whole study area with yearly stage-specific CMM incidences directly standardised according the European age-standard population [18] in order to facilitate comparative analyses with data from other European countries.

#### Disease mapping

Disease mapping was performed using the Rapid Inquiry Facility (RIF) 4.0 [19, 20] to produce maps showing spatial variations in the stage-specific incidences of CMMs. Regional maps were created to visualise spatial disparities in indirectly standardised incidence ratios (SIRs) within the whole study area, while local maps were used for the two urban areas considered. The comparison area was set to the whole study area in the regional mapping and the corresponding urban area in the local studies. While a regional map may indicate systematic differences in SIRs between different urban areas, a local map can convey SIR variations *within* an urban area. The SIRs were derived from the observed and expected numbers of stage-specific cases in each small-area. The expected numbers were calculated based on incidences stratified by sex and age group (30–34, 35–39,…, 85–89, 90 + years of age) in the comparison population [19]. To reduce the influence of the small counts, we applied a Bayesian smoothing of the SIRs [21] according to the Besag-York-Mollié (BYM) model [22] built into the RIF 4.0 [19].

For a given stage-specific CMM outcome, $\hat{\theta }$_*i*_ denoted the estimated SIR in small-area *i* (*i* = 1, 2,…, *n*) and PP denoted the corresponding posterior probability of SIR > 1. PP is a measure influenced by the point estimate $\hat{\theta }$_*i*_ and its uncertainty [23]. Based on this measure, we defined ‘signals’ as small-areas with more or less pronounced posterior probability of an elevated or lowered smoothed standardised incidence. We considered four cut-offs for the posterior probabilities:

- PP > 0.90, coloured in red (a strong signal indicating an elevated incidence);
- 0.90 ≥ PP > 0.80, coloured in light red (a moderate signal indicating an elevated incidence);
- 0.80 ≥ PP > 0.20, coloured in yellow;
- 0.20 ≥ PP > 0.10, coloured in light green (a moderate signal indicating a lowered incidence);
- 0.10 ≥ PP, coloured in green (a strong signal indicating a lowered incidence).

Two types of errors can be associated with such signals: (i) false-positive signals, indicating a small-area having an elevated or lowered incidence when in fact its underlying true rate equals the average level in the comparison area (lack of specificity); and (ii) false-negative signals, indicating a small-area to be in the average incidence level when in fact its underlying rate is elevated or lowered (lack of sensitivity). The 0.80 and 0.90 cut-offs for the ‘elevated incidence’ signals were chosen based on a comprehensive simulation study previously published [23]. We used analogous cut-offs (0.20 and 0.10) for the ‘lowered incidence’ signals. To our knowledge, no simulation study has investigated such ‘two-sided’ scenarios to identify optimal cut-off values.

#### Sensitivity analyses with conclusions about model choice

We conducted three sets of sensitivity analyses to (i) assess the impact of default prior distributions used for the Bayesian smoothing; (ii) assess the need to account for zero-inflation in the model; and (iii) visually assess the performance of our detection of signals compared to that of the standard scan statistics cluster detection method. The results on sensitivity are presented as Supplementary material. In the following four paragraphs, we comment upon the results and convey our conclusion about model choice.

The BYM model requires prior distribution for the parameters. By default, the RIF 4.0 software specifies minimally informative priors on the logs of both the unstructured and structured effect precision. This first sensitivity analysis showed that changing the priors made no significant difference to the smoothed SIR in the disease mapping results (Supplementary Table S1).

Each disease mapping was based on a data set consisting of the observed and expected numbers of cases in each small-area *i* (*i* = 1, 2,…, *n*). In a context where this data set contains many zero observations and small expected numbers, the BYM model based on a Poisson distribution may be less appropriate than a zero-inflated distribution [24]. This second sensitivity analysis, based on the Deviance Information Criteria [25] showed that the standard Poisson distribution provided a better fit for these studies with low incidence counts than the two zero-inflated alternatives considered (Supplementary Table S2).

Scan statistics are often used in cluster detection [26]. The comparison of the two methods suggested a good agreement for the Stage I CMM maps, but highlighted some differences for stage III-IV, possibly attributable to specific parametrisation of the methods (Supplementary Fig. S2).

In conclusion, the sensitivity analyses provided support for using the BYM model built into the RIF 4.0.

#### Associations with small-area deprivation

We employed ecological regression for estimating associations between small-area deprivation (i.e., small areas grouped according to deprivation quintiles) and stage-specific CMM incidences. By standard generalized linear regression procedures, we modelled associations between deprivation quintile, represented as a categorical variable, and logarithm of smoothed SIR, *ln*($\hat{\theta }$_*i*_). Inverse variances of *ln*($\hat{\theta }$_*i*_) were incorporated as weights in the regression analyses. In addition to the association estimates, we present box-plots of the distributions of smoothed SIRs *within* each quintile of deprivation.

## Results

### Analysis of proportion of cases with late-stage tumours

Of the 10,607 cases that were retrieved, 300 had insufficient data to assess clinical stage and 5 could not be geocoded leaving an analytic sample of 10,302 CMM cases. They comprised 7302 stage I (70.9%), 2273 stage II (22.1%) and 727 stage II–IV (7.1%) cases. The proportion of CMM cases with stage II–IV tumours increased from 14% in the 30–35 years old to 68% in the 90 + years old (Table 1). The proportion of CMM cases with stage III–IV disease varied between 5 and 9% across the age groups (Table 1).

The proportion of CMM cases with stage II–IV tumours was significantly lower in residential small-areas belonging to the wealthiest quintile than those in the poorest quintile (Table 1). The corresponding odds ratios (ORs) were less pronounced within the whole study region (OR 1.40) than in the two urban areas (Gothenburg, OR 2.95; Malmoe, OR 2.71). From these results one may infer that late-stage CMMs were much more common in deprived areas; however, this conclusion is incorrect (see below). The corresponding proportions of CMM cases with stage III–IV tumours tended to be lower in small-areas belonging to the wealthiest quintile than those in the poorest quintile, although less markedly than by including stage II CMMs in the late-stage group (Table 1).

### Analysis of stage-specific incidences

#### Overall time-trends

**Fig. 1:**
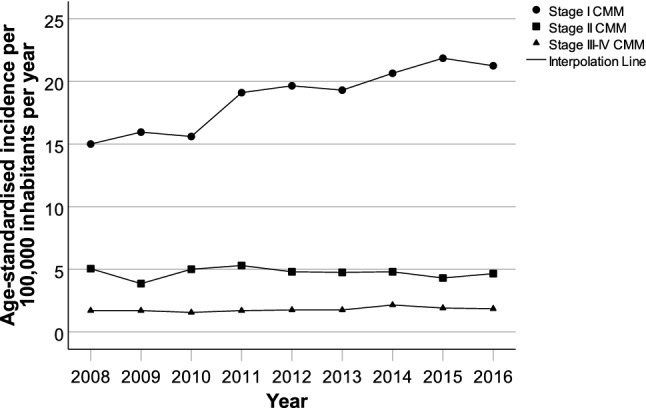
Time trends of stage I, stage II and stage III-IV CMM incidences in South-West Sweden, 2008–2016. The yearly, age-standardised incidences (by using the European standard population) are shown

Figure 1 shows the overall, age-standardised incidences of stage I, II and III–IV CMMs, respectively. Early-stage CMM showed an increasing incidence trend in the period 2008–2016, from 15 to 22 per 100,000 inhabitants per year. The incidences of stage II and III-IV CMMs remained relatively stable in the study period, being approximately 5 and 2 per 100,000 inhabitants per year, respectively.

#### Disease mapping

**Fig. 2:**
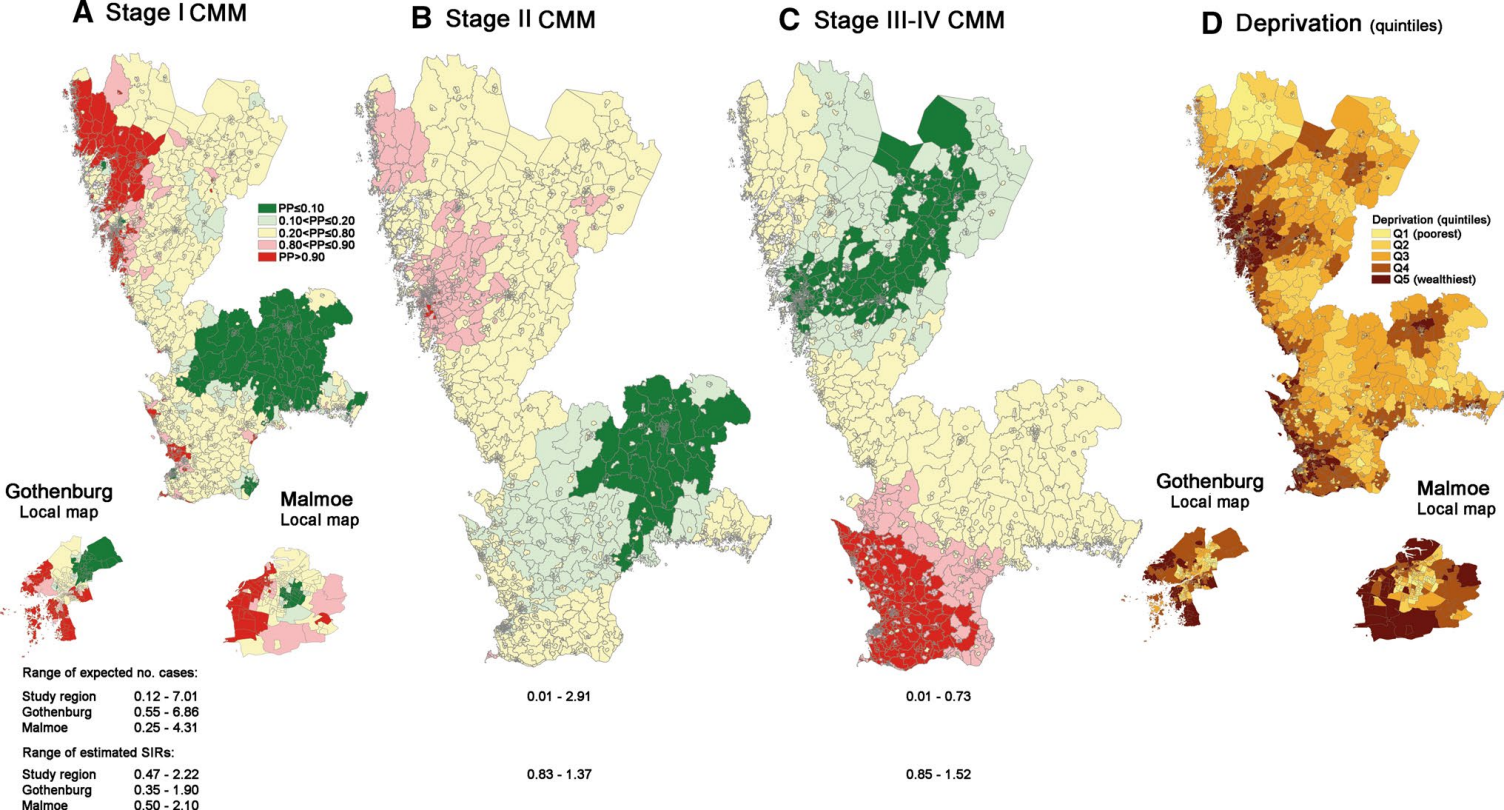
The regional and local maps visualising spatial disparities in **a** stage I, **b** stage II and **c** stage III-IV cutaneous malignant melanoma (CMM) in the Southern and Western Swedish Health Care Regions. Red, light red, green and light green coloured DSAs reflect signals of heterogeneity based on the posterior probability of standardised incidence ratio > 1 (PP)*.* The local maps of Gothenburg and Malmoe are not shown in **b** and **c** because of absent signals of spatial heterogeneity for stage II and stage III-IV CMMs, respectively, within these local areas. **d** Descriptive map of small-area deprivation

Figure 2a presents the regional and local maps visualising spatial inequalities in stage I CMM incidence. Out of the 2173 small-areas in the regional map, 341 (15.5%) showed strong signals of elevated stage I incidence and 296 (13.6%) strongly signalled a lowered stage I incidence. The proportion of variance explained by the structured spatial component of the BYM model used was 0.96, suggesting that approximately 96% of the variability can be explained by the spatial structure [24]. Figure 2 provide information about ranges of (i) expected number of cases and (ii) smoothed SIRs across the small areas for each map. For the regional map of stage I CMM, it is shown that expected number of cases ranged between 0.12 och 7.01 and the smoothed SIRs vary between 0.47 and 2.22. Clusters of higher than expected cases were revealed in the northwest of the study area and in several coastal areas (for geography, see Supplementary Fig. S1). Clusters of DSAs with lower than expected cases were observed in a widespread mid-east area of the study region and in a few smaller areas, including a cluster in the Malmoe area. The local maps of Gothenburg and Malmoe revealed clear clusters of excess or deficit incidences within the two urban areas. Within Gothenburg, 58 and 56 DSAs (out of 306) strongly indicated elevated and lowered stage I incidence respectively. Within Malmoe, the corresponding numbers of small-areas with elevated and lowered stage I CMM incidences were 39 and 32, respectively (out of 192 DSAs).

**Fig. 3:**
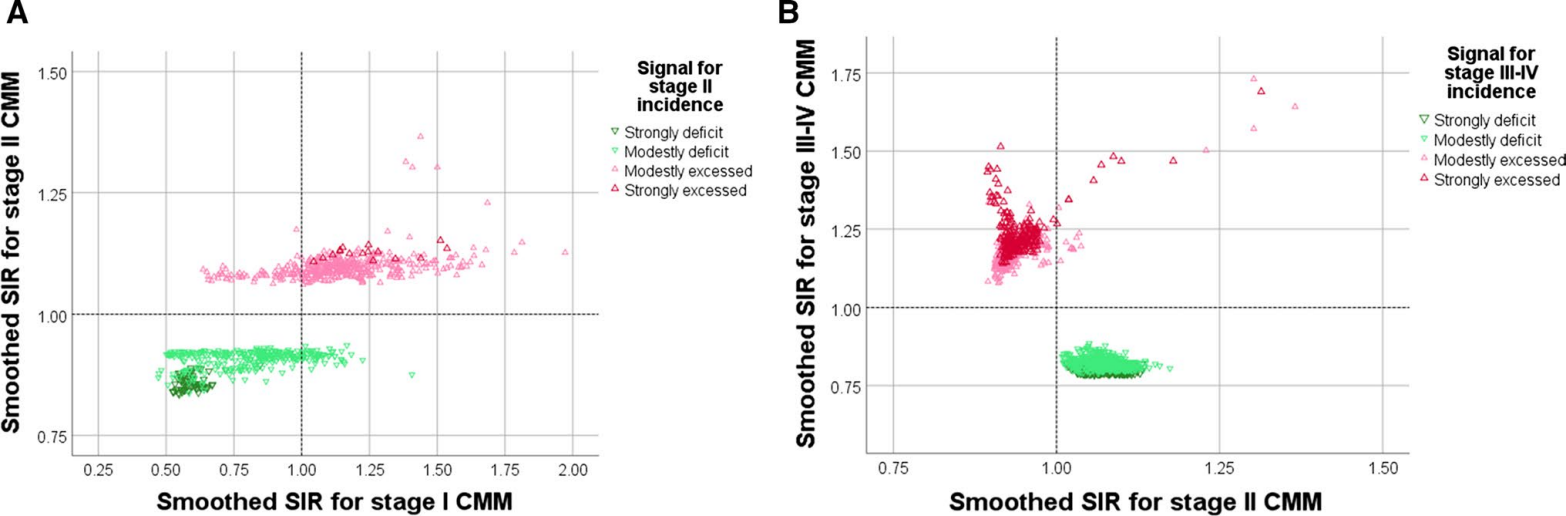
Associations between the spatially smoothed standardised incidence ratios (SIRs) for **a** stage I versus stage II cutaneous malignant melanoma (CMM), considering only the DSAs that indicated excess or deficit incidence of stage II CMM, and **b** for stage II versus stage III-IV cutaneous malignant melanomas (CMMs), considering only the DSAs that indicated excess or deficit incidence of stage III–IV CMM

The stage II maps revealed more uniform patterns than the stage I maps. Figure 2b shows the regional map of stage II CMM with smoothed SIRs ranging between 0.83 and 1.37. The local maps are not shown as we did not find any signals of elevated or lowered stage II incidence, neither within Gothenburg nor within Malmoe. When comparing the elevated/lowered stage II signals with stage I CMM incidence, we found that (i) 342 out of 412 (84%) small-areas with elevated stage II CMM incidence also showed a SIR > 1 for stage I CMM and (ii) 336 out of 390 (85%) small-areas with deficit stage II incidence also showed a SIR < 1 for stage I CMM (Fig. 3a).

The regional stage III–IV map revealed two large clusters of elevated or lowered stage III–IV incidence (Fig. 2c). The local maps are not shown because there was no signals of spatial heterogeneity. The cluster of higher than expected stage III–IV cases appeared in the southwest of the study region, in which 249 small-areas strongly signalled an elevated stage III–IV incidence. The cluster of lower than expected stage III–IV cases appeared along a widespread area from Gothenburg to the north-eastern part of the study region, in which 308 small-areas showed a strong signal of lowered stage III–IV incidence. When comparing the elevated/ lowered stage III–IV signals with stage II CMM incidence, we found that (i) 663 out of 687 (97%) small-areas with elevated stage III–IV CMM incidence showed a reversed SIR < 1 for stage II CMM and (ii) all small-areas with deficit stage III-IV incidence (*n* = 882) showed a reversed SIR > 1 for stage II CMM (Fig. 3b).

#### Associations with small-area deprivation

The stage I CMM incidence was lower in more deprived small-areas (Table 2, Supplementary Fig. S4). Within the whole region, 43% (143/341) of the DSAs that showed strong signals of elevated stage I incidence belonged to the wealthiest quintile, whereas 41% (120/296) of the small-areas that strongly signalled a lowered stage I incidence belonged to the poorest quintile. The corresponding trends within Gothenburg and Malmoe revealed more marked gradients. A twofold higher average-level stage I incidence was estimated in Q5 (wealthiest quintile) compared to Q1 (poorest quintile) in Gothenburg as well as in Malmoe (Table 2). Within Gothenburg, 64% (37/58) of the DSAs that showed strong signals of elevated stage I incidence belonged to the wealthiest quintile and 64% (36/56) of the small-areas that indicated deficit stage I incidence belonged to the poorest quintile. Within Malmoe, 49% (19/39) of the strongly signalled elevated stage I CMM small-areas belonged to the wealthiest quintile and 69% (22/32) of the deficit early-stage CMM small-areas belonged to the poorest quintile.

**Table 2:** Associations between small-area deprivation and stage-specific cutaneous malignant melanoma (CMM) incidences, estimated from ecological regressions

	Stage I CMM		Stage II CMM	
	Ratio between average-level incidences	(95% CI)	Ratio between average-level incidences	(95% CI)
Small-area deprivation (median income) quintiles within the study region				
Q1	1.0	Ref.	1.0	Ref.
Q2	1.11	(1.08–1.15)	1.01	(1.00–1.02)
Q3	1.11	(1.08–1.15)	1.01	(0.99–1.02)
Q4	1.18	(1.14–1.21)	1.02	(1.00–1.03)
Q5 (wealthiest)	1.35	(1.31–1.39)	1.04	(1.03–1.05)
Small-area deprivation (median income) quintiles within Gothenburg				
Q1	1.0	Ref.	^a^	
Q2	1.39	(1.27–1.53)	^a^	
Q3	1.43	(1.31–1.57)	^a^	
Q4	1.55	(1.42–1.70)	^a^	
Q5	1.95	(1.78–2.13)	^a^	
Small-area deprivation (median income) quintiles within Malmoe				
Q1	1.0	Ref.	^a^	
Q2	1.26	(1.12–1.43)	^a^	
Q3	1.45	(1.28–1.65)	^a^	
Q4	1.65	(1.46–1.86)	^a^	
Q5	2.01	(1.77–2.27)	^a^	

Small-area deprivation is defined by median income quintiles in thousands of Swedish krona per year independently for each of the three study areas considered: the Southern and Western Swedish Health Care Regions, and the municipalities of Gothenburg and Malmoe. Q1: Poorest areas. Q5: wealthiest areas

^a^No signals of excessed/deficit Stage II incidence were found within Gothenburg and Malmoe, respectively

The increasing trend of smoothed SIRs across the quintiles of small-area deprivation within the study region, which we observed for stage I CMM, was gradually attenuated for later stages of CMM (Supplementary Fig. S3). Stage II CMM showed a marginally increasing trend across the median income quintiles (Table 2), while stage III–IV CMM showed no such trend.

## Discussion

Our objective was to demonstrate a relatively simple approach based on disease mapping and trends analysis to explore patterns of incidence of CMM in relation to stage of diagnosis and deprivation in regional and local areas in the South-West of Sweden. Extensive work on producing cancer incidence atlases have been carried out in many countries including the UK and the Nordic countries [7, 27]. Such systems for providing spatially related cancer information have focused on incidences regardless of disease stage at diagnosis. To our knowledge, no geographical information system has routinely been linking cancer incidence data per stage at diagnosis and only a few studies have addressed disease mapping of stage-specific cancer incidences [28–31].

Our analysis showed an increasing time trend of stage I CMM, but a stable stage II–IV CMM incidence, in the study region over the nine-year observation period (2008–2016). We cannot infer that early detection has been improving in the study region, because of the absence of reductions in the stage II–IV CMM incidences—which should be of primary concern. Monitoring spatio-temporal trends in early detection can help assess progress after the launch of screening programmes. Furthermore, our analysis showed that stage I CMM incidence was markedly higher in wealthier small-areas, in particular within each urban area. A twofold higher stage I incidence was observed, on average, in the wealthiest small-areas (upper quintile) than in the poorest small-areas (lower quintile). We identified in the regional map of stage III–IV CMM two clusters of higher or lower than expected late-stage incidences which were quite distinct from those identified for stage I.

### Strengths and limitations

Our study includes a large number of CMM cases from the South-West of Sweden, in which lives a third of the Sweden national population. The Cancer registry data used has excellent coverage. The small-area units used in this study have been recently developed, providing new scope to assess spatial disparities.

By using small-area data, our analysis provides more granularity than in previous studies. For example, previous CMM maps based on crudely defined geographical entities, i.e. municipalities (of which there are 99 in the Southern and Western Swedish Health Care Regions, compared with 2173 DSAs) and urban districts (there are 10 districts in Gothenburg, compared with 306 DSAs, and 10 districts in Malmoe, compared with 192 DSAs) [30] largely masked heterogeneities in social deprivation. Correlations between stage-specific CMM incidences and small-area deprivation could therefore not be addressed in a meaningful way.

As in other types of ecological studies, the ecological fallacy is inherent in small-area analyses [32]. The small-area design attempts to minimise this potential bias by using small geographies that provide a closer estimation to individual-level risks. Notwithstanding the ecological fallacy, small-area analyses are useful for drawing inference beyond individual-level risks [33].

We did not analyse spatio-temporal trends. We have previously analysed time trends in different population groups according to sex, educational level and immigrant status, and the results indicated similar temporal trends across those groups (i.e., increasing early-stage CMM incidence and stable late-stage CMM incidences) [34].

A validated deprivation index was lacking in our analysis. A small-area multiple index of deprivation, such as the English Index of Multiple Deprivation (32,844 small areas) [35, 36] or the Scottish Index of Multiple Deprivation (6505 small areas) [37], would have strengthen our approach. There are extensive requirements for creating such a small-area deprivation index [38], and various proxys of deprivation are available in different countries. To our knowledge, such an index is nevertheless not currently available for Sweden.

### Policy implications

Monitoring inequalities in early detection may support targeted efforts to improve early detection in disadvantaged areas. Geographical targeting at area-level may be a feasible approach, e.g. by involving the primary health care in the targeted small-areas, and the subsequent elimination of inequalities in stage at diagnosis may result in substantial reductions in deaths within 5 years of a diagnosis [39]. If actions for detecting melanoma are considered, one should take into account that screening initiatives might amplify a risk of ‘overdiagnosis’ by increased detection of slow-growing, thin, non-metastasizing CMMs. The most common histologic subtype of CMM, superficial spreading melanoma, is relatively slow-growing and has an in situ growth phase before becoming invasive, whereas the second most common type, nodular melanoma, is fast-growing and usually invasive at diagnosis [40]. Elsewhere, screening programs for CMM in the general population have implied rising incidences, but not turned out to be efficient, in terms of reducing mortality, for example in Germany [41] and other countries [42]. The marked association between elevated stage I CMM diagnosis and lower small-area deprivation shown in our results were probably affected by disparities in peoples’ awareness and demand on health. Within each urban area (Gothenburg and Malmoe), access to primary health care and specialist care should be consistent, similarly, pathologists’ classifications of early-stage CMMs should not differ systematically as similar diagnostic routes are applied.

The approach used here could be adopted by National Cancer Registers to develop routines for geo-coding incident cancer cases into adequately small areas, linked with socio-demographic characteristics. Such developments could facilitate the monitoring of spatial and social inequalities in incidence and early detection of cancer.

### The need for reliable and relevant data

The proposed method depends on the availability of high spatial resolution data on cancer stages and deprivation. In Sweden, stage data are routinely collected by the National Quality Registries, with excellent coverage for many cancer types [13]. There is a growing push across European countries to collect and make available reliable stage at diagnosis data collected by national cancer registries to help estimate stage-specific incidence and survival. A recent assessment of the cancer stage data from 62 registries concluded that the completeness of primary data and the accuracy of stage coding needed to be improved for cancer registries to fulfil their role in cancer control [43]. Nevertheless, accessing such data at individual level to enable linkage with other data sources requires strict information governance and approval by data providers. It is then possible to use small areas, with accessible socio-demographic data, for disease mapping [44].

## Conclusion

Our analysis of CMM supported the use of the cancer stage incidence mapping method for revealing geographical and sociodemographic inequalities in cancer detection. It should be of interest to apply the method in other settings, for monitoring inequalities in early detection of CMM as well as other common cancer types (e.g. breast, prostate, colorectal and lung cancer). Further analyses will yield more firm conclusions about the suggested analytic approach, “cancer stage mapping”, and its potential implications.

## Electronic supplementary material

Below is the link to the electronic supplementary material.

Supplementary file1 (PDF 1100 kb)
