# Supplementary material for: Disease mapping of early- and late-stage cancer to monitor inequalities in early detection: a study of cutaneous malignant melanoma
Source: Eur J Epidemiol. 2020 Apr 30;35(6):537–47. doi: 10.1007/s10654-020-00637-0 (PMC7320924; doi:10.1007/s10654-020-00637-0)
Supplement: Supplementary file 1 — Supplementary file1 (PDF 1100 kb) [file 10654_2020_637_MOESM1_ESM.pdf]

Supplementary material

Disease mapping of early- and late-stage cancer to monitor inequalities in early detection: a study of cutaneous malignant melanoma

Supplementary Figures

Supplementary Figure S1

Overview of the study areas. The Southern and Western Swedish Health Care Regions are shown in blue, with the boundaries of the 2,173 Demographic Statistics Areas (DSAs) - or neighbourhoods - displayed. The mean population size (2008-2016) of DSAs in the regions ranged between 600 and 2,600. The extent and DSAs for the two main municipalities of the regions are shown in green. Gothenburg is divided into 306 DSAs and had a population aged ≥30 years of 0.34 million in 2016. There are 192 DSAs in Malmoe, which had a population aged ≥30 years of 0.20 million. Each case of cutaneous malignant melanoma was geocoded to a DSA.

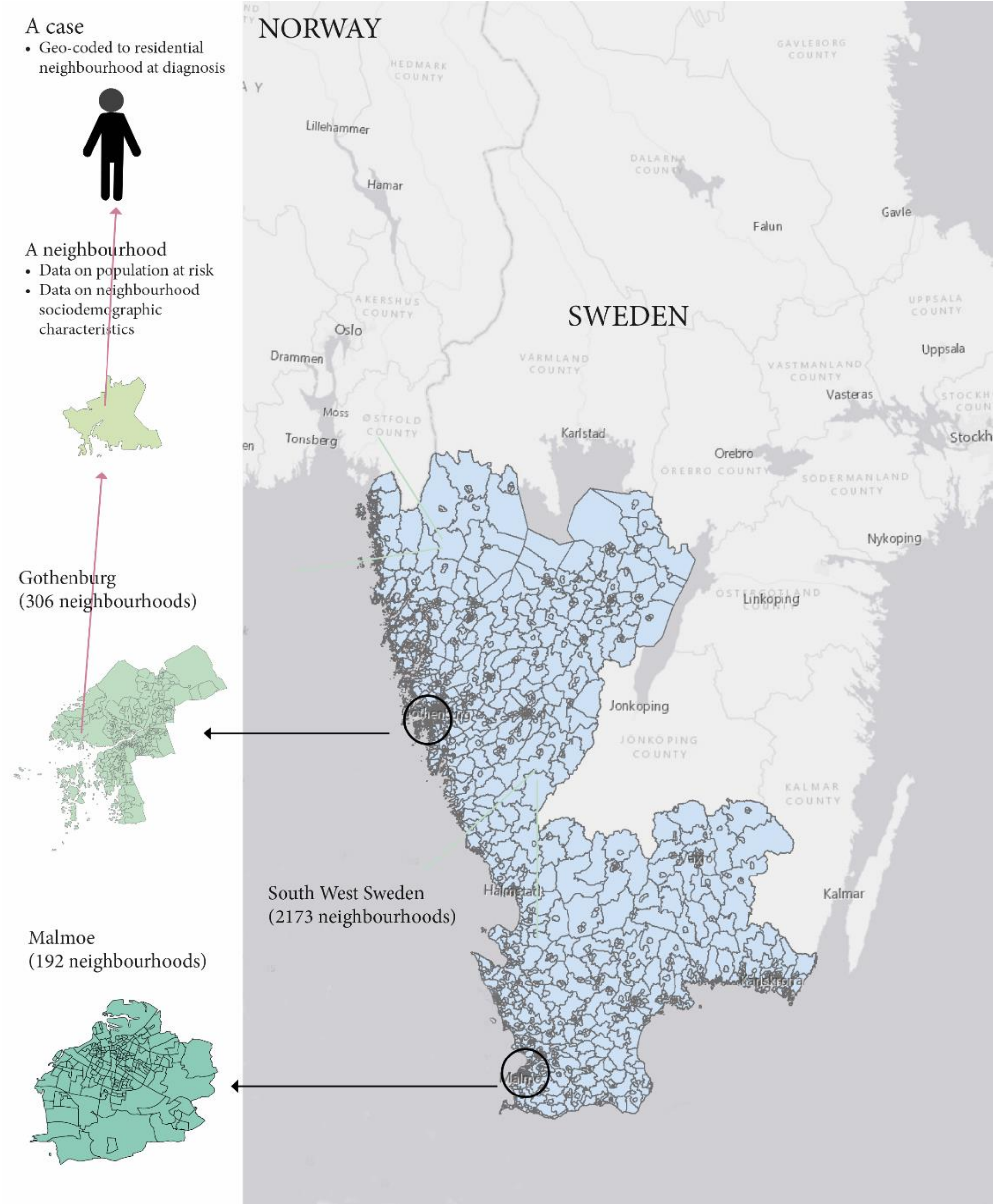

Supplementary Figure S2

Regional maps of the Southern and Western Swedish Health Care Regions showing a 'heat map' of likely cluster locations created using expectation-based Poisson scan statistic [scanstatistic *R* library, 2018] on the Stage I and Stage III-IV CMM incidence data and output of disease mapping using Bayesian smoothing. In the Result section of the manuscript, areas of higher than expected cases are identified as 'clusters' from visual inspection of the disease maps of the posterior probability from the Bayesian smoothing methods. While the primary purpose of this paper is to investigate inequalities in early detection of cancer, it is worth comparing the disease maps presented in Fig. 2 with methods specifically designed for the detection of spatial disease clusters. Using the output from the Stage 1 CMM and Stage III-IV studies we ran expectation-based Poisson scan statistic [scanstatistic *R* library, 2018], the results are shown Fig. S3. Comparison of Fig. S3 (A) with Fig. 2 (A), the areas of high posterior probability overlap well for the Stage I CMM maps. However for the Stage III-IV maps (Fig. S3 (B) and Fig. 2 (C)) the large cluster of higher than expected stage III-IV cases in the southwest of the study region (Fig. 2 (C)) is not fully represented in the output of the cluster analysis (Fig. S3(B)). This could be due to the size of the area of higher than expected stage III-IV cases which covers 249 neighbourhoods which is larger than the maximum size of the zones of nearest neighbours that the scan statistic software was configured to use (200). It should also be noted that there are several different methods of identifying spatial clusters of non-infectious diseases, but a full comparison of these methods with our results was beyond the scope of this paper.

A. Stage 1 CMM

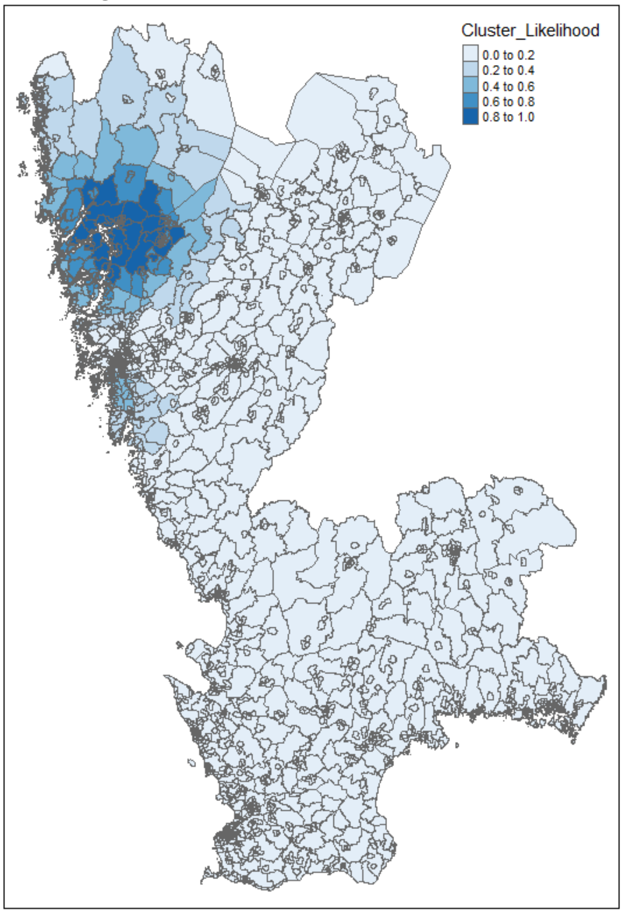

B. Stage III-IV CMM

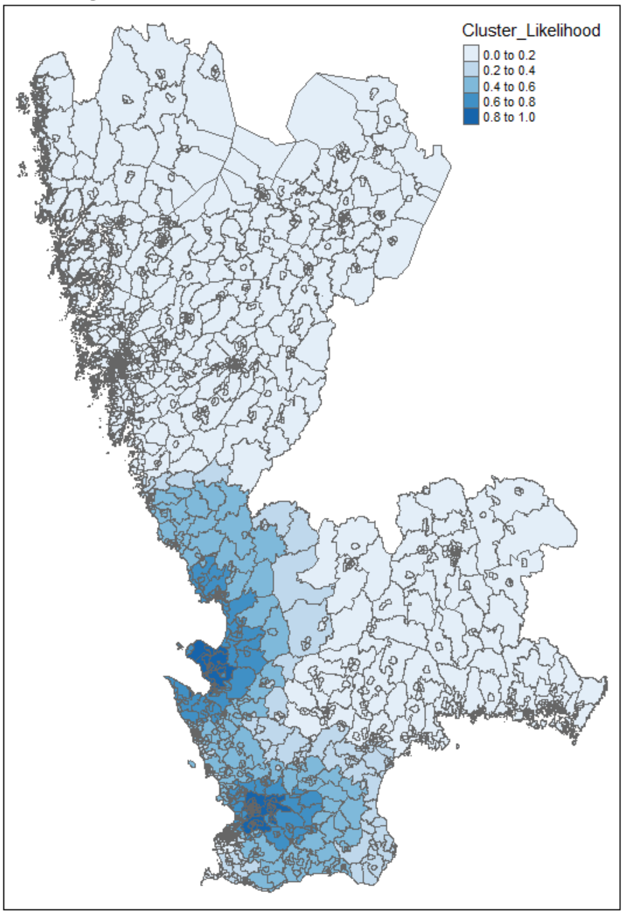

Supplementary Figure S3

Associations between neighbourhood deprivation and the spatially smoothed standardised incidence ratios (SIRs) of stage I, stage II and stage III-IV cutaneous malignant melanomas (CMM) within the Southern and Western Swedish Health Care Regions (A, D and E) and the municipalities of Gothenburg (B) and Malmoe (C). Deprivation quintiles are based on the median income per household per consumption unit. The box-plots were produced by using IBM SPSS Statistics for Windows version 25.0 (IBM Corp.: Armonk, NY, USA).

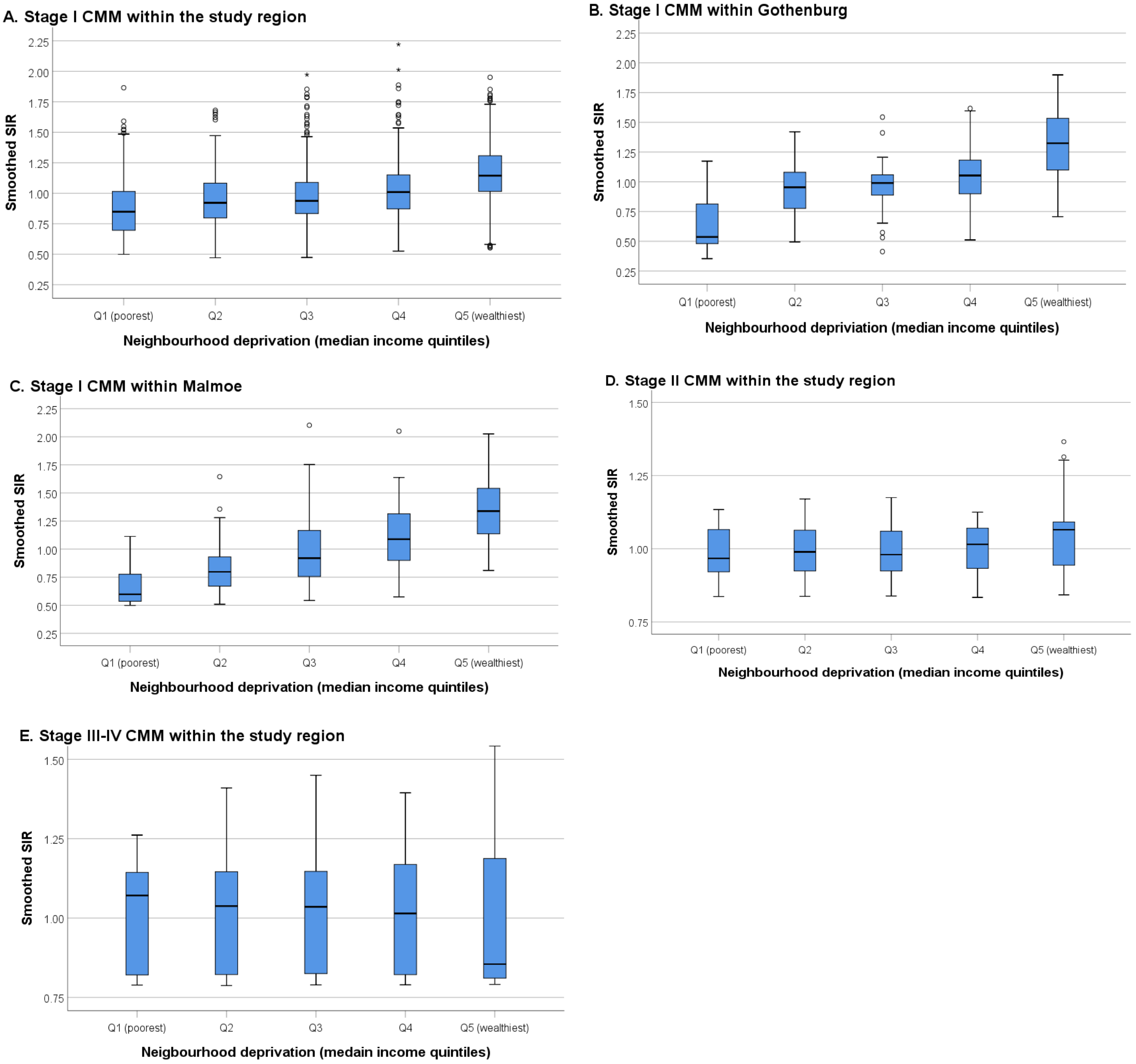

Supplementary Tables

Supplementary Table S1

Summary of results of using different prior distributions for the unstructured and structured effect precision of the BYM model. By default, the RIF 4.0 software specifies minimally informative priors on the logs of both the unstructured and structured effect precision ($\log(\tau v)$ ~ logGamma(0.5, 0.0005) and $\log(\tau u)$ ~ logGamma(0.5, 0.0005) respectively). We performed sensitivity analyses to investigate the impact on the smoothed SIR of changing the priors for the model aimed at estimating the spatial variations in the Stage 1 CMM incidence within the study area (South-West Sweden). The results of the sensitivity analysis show that changing the priors made no significant difference to the smoothed SIR in the disease mapping results:

Prior distributions for unstructured and structured effect precision	Mean absolute difference in smoothed SIR from main model (logGamma(0.5, 0.0005))	Maximum absolute difference in smoothed SIR from main model (logGamma(0.5, 0.0005))	Deviance Information Criteria (DIC)[a]
**logGamma(0.5, 0.0005) (main model – default)**	N/A	N/A	8692.4
**logGamma(1.0, 0.0005)**	0.0013	0.0089	8692.3
**logGamma(1.0, 0.001)**	0.0008	0.0051	8692.3
**logGamma(1.0, 0.01)**	0.0049	0.0346	8692.8
**logGamma(0.5, 0.00001)**	0.0012	0.0084	8692.2
**Flat**	0.0022	0.0160	8692.2
**Normal (0,1)**	0.0013	0.0089	8695.9

[a] Spiegelhalter et al, 2002.

Supplementary Table S2

Deviance Information Criteria comparing Poisson with Zero Inflated Poisson distributions for Stage III-IV CMM incidences across the South-West Sweden region (BYM model). The incidence data for the late stage CMM (Stage III-IV) contains a large number of zeros across the neighbourhoods. While these zeros are all sampling zeros (i.e. there is no structural reason why so many neighbourhoods have zero counts), it may be that using a zero-inflated Poisson distribution of either type 1 (ZIP1 – assumes structural and sampling zeros in the data) or 0 (ZIP0 – assumes only structural zeros in the data) instead of the default Poisson distribution will prove a better fit for the data [Agarwal et al, 2002]. We performed sensitivity analyses on the model that mapped the Stage III-IV CMM incidences across the South-West Sweden region. The results of the sensitivity analysis show that the Poisson distribution gives the lowest DIC, suggesting it is the better model selection compared to the ZIP1 and ZIP0 distributions:

Distribution	Deviance Information Criteria (DIC)[a]
**Poisson**	3121.3
**ZIP1**	3122.8
**ZIP0**	3217.3

[a] Spiegelhalter et al, 2002.

References in Supplementary material section

Agarwal DK, Gelfand AE, Citron-Pousty S. Zero-inflated models with application to spatial count data. Environ Ecologic Statist. 2002;9:341-55.

scanstatistics: Space-Time Anomaly Detection using Scan Statistics. Author: Allévius B, 2018. https://cran.r-project.org/web/packages/scanstatistics/index.html Accessed 1 Feb. 2020.

Spiegelhalter DJ, Best NG, Carlin BP, Van Der Linde A. Bayesian measures of model complexity and fit. J R Statist Soc B. 2002;64:583-639.
